# Unravelling the link between chronic inflammation and primary hyperparathyroidism: a systematic review

**DOI:** 10.3389/fimmu.2025.1563967

**Published:** 2025-06-02

**Authors:** Ana Gheorghe-Milea, Oana Stănoiu-Pînzariu, Carmen Emanuela Georgescu

**Affiliations:** ^1^ Department of Endocrinology, Iuliu Haţieganu University of Medicine and Pharmacy, Cluj-Napoca, Romania; ^2^ Endocrinology Clinic, Cluj County Emergency Clinical Hospital, Cluj-Napoca, Romania

**Keywords:** chronic inflammation, inflammatory biomarkers, primary hyperparathyroidism, bone resorption, cardiovascular disease

## Abstract

**Introduction:**

Primary hyperparathyroidism (PHPT) is a multisystemic endocrine disorder characterized by an incompletely understood pathogenesis, a complex clinical picture and various complications. Chronic inflammation represents a state that can affect the normal function of cells and cause tissue damage, therefore increasing the risk of certain diseases, including cancer, metabolic, cardiovascular or neurodegenerative disorders.

**Aim:**

Reviewing existing data on markers of inflammation in patients with PHPT, with potential implications in understanding the pathogenesis of PHPT, stratifying the risk for complications and providing new diagnostic biomarkers and a personalized therapeutic approach, especially in patients who cannot be operated on.

**Methods:**

A systematic review was conducted by searching in four electronic databases (PubMed, Embase, Web of Science and Scopus) and summarizing data from studies that evaluated inflammatory markers in patients with PHPT.

**Results:**

The review included a total of 28 articles, encompassing data from 1572 patients diagnosed with PHPT. Various markers associated with chronic inflammation, including High sensitivity C-Reactive Protein (CRP), Tumor Necrosis Factor-α, Interleukin (IL)-6, and fibrinogen, were found to be elevated in PHPT patients. White blood count (WBC) values were similar in patients and controls in most studies, while for some markers derived from the full blood count significant differences were found between these groups. Correlations between PTH levels and several biomarkers, including IL-6, CRP and WBC, were also identified. Data on the impact of parathyroidectomy on inflammation parameters were conflicting.

**Conclusion:**

The findings from this systematic review suggest an association between chronic inflammation and primary hyperparathyroidism, underscoring the potential role of inflammation as a mediator of PHPT-related complications. Targeting inflammatory pathways may offer novel therapeutic strategies for mitigating systemic effects of PHPT and improving patient outcomes.

## Introduction

Primary hyperparathyroidism (PHPT) is a multisystemic endocrine disorder traditionally defined by hypercalcemia and inappropriately elevated parathyroid hormone (PTH) levels; however, a subset of patients displays normal calcium levels, being classified as normocalcemic PHPT (1). PTH plays a key role in the regulation of calcium and phosphate metabolism, increasing calcium reabsorption by the kidneys, stimulating the renal activation of vitamin D, while also inhibiting phosphate reabsorption from the tubules. The abnormal secretion of PTH is usually caused by a single parathyroid adenoma (80% of cases), but parathyroid hyperplasia and parathyroid carcinoma can also account for 15-20% and <1% of cases, respectively (2). PHPT ranks as the third most prevalent endocrine disorder, following diabetes and thyroid conditions (3). Women are predominantly affected, with a peak incidence in the 5th and 6th decades (3). The widespread use of routine serum calcium measurements has resulted in higher reported incidence and prevalence of PHPT (4). Although classically this condition has been characterized by the phrase “stones, bones, groans, and moans” (5), the complexity of the clinical picture of PHPT has been recognized in recent years, as numerous patients can be asymptomatic at diagnosis or present with non-traditional features such as neuromuscular, cognitive, cardiovascular (CV) or metabolic complications of PHPT (6).

Chronic inflammation can alter immune tolerance, potentially disrupting normal cellular function and causing tissue damage, therefore increasing the risk of certain diseases, including cancer, metabolic, CV or neurodegenerative disorders (7–10). Consequently, chronic inflammation markers including Interleukin (IL)-6, Tumor Necrosis Factor (TNF)-α, fibrinogen or High sensitivity C-Reactive Protein (Hs-CRP) have emerged as potential instruments for CV risk stratification (11, 12). While the most common inflammatory markers evaluated in the clinical practice include C-Reactive Protein (CRP), erythrocyte sedimentation rate (ESR), procalcitonin, and white blood count (WBC), in recent years a set of biomarkers that can be directly obtained or calculated from the full blood count (FBC) have been evaluated in relation to several diseases (13–15). These include the neutrophil to lymphocyte ratio (NLR), the platelet to lymphocyte ratio (PLR), the lymphocyte to monocyte ratio (LMR), red blood cell distribution width (RDW), the monocyte-to-high-density lipoprotein cholesterol ratio (MHR), systemic immune-inflammation index (SII)- calculated as the product of platelet and neutrophil counts divided by the lymphocyte count or the systemic inflammation response index (SIRI)- the product of neutrophil and monocyte counts divided by the lymphocyte count. Cytokines are a family of molecules with important implications in regulating immune cell function, as well as other processes outside the hematopoietic environment. Some cytokines act as proinflammatory molecules (IL-6 and 8, TNF-α/β, IL-1α/β, or Interferon-α/γ), while others have an anti-inflammatory effect (soluble IL-1 receptor, TNF-α binding protein, adiponectin or IL-10) (16).

Evidence from the literature indicates a possible link between PTH levels and inflammation. For example, Cheng et al. (17) identified a significant association between PTH levels and CRP, RDW and PLR in the general US population and Yang et al. (18) postulated that these findings could indicate that hyperparathyroidism, either directly or as a result of abnormal calcium and phosphate metabolism, might be responsible for the development of systemic inflammation and its related complications. A series of studies, most of them performed on lymphocytes from patients with secondary hyperparathyroidism, revealed that hypersecretion of PTH might impair the cellular immune response (19, 20). Moreover, the presence of lymphocyte infiltrates in parathyroid adenomas was correlated with higher levels of PTH, potentially indicating a cytokine mediated change in the endocrine activity of parathyroid cells (21).

The aim of this paper was to review and analyze existing data on markers of inflammation in patients with PHPT with potential implications in understanding the pathogenesis of PHPT, stratifying the risk for complications and providing new diagnostic biomarkers and a personalized therapeutic approach, especially in patients who cannot be operated on.

## Methods

A systematic review was conducted until the 10^th^ of April 2025, interrogating four electronic databases (PubMed, Embase, Web of Science and Scopus). The search strategy included one term related to PHPT (“primary hyperparathyroidism”, “parathyroid adenoma” or “parathyroid neoplasm”) and one related to chronic inflammation (“inflammation”, “chronic inflammation”, “systemic inflammation”, “persistent inflammation”, “long-term inflammation”, “inflammatory markers”, “inflammatory mediators”, “biomarkers of inflammation”, “pro-inflammatory markers”, “cytokine”, “pro-inflammatory cytokines”, “anti-inflammatory cytokines”, “tumor necrosis factor”, “TNF”, “interleukins”, “IL-1”, “IL-6”, “IL-8”, “IL-10”, “IL-17”, “IL-17A”, “C-reactive protein”, “CRP”, “MCP-1”, “chemokine”, “inflammatory response”, “immune response”, “oxidative inflammation”, “inflammatory cascade” or “immune activation”).

**Figure 1:**
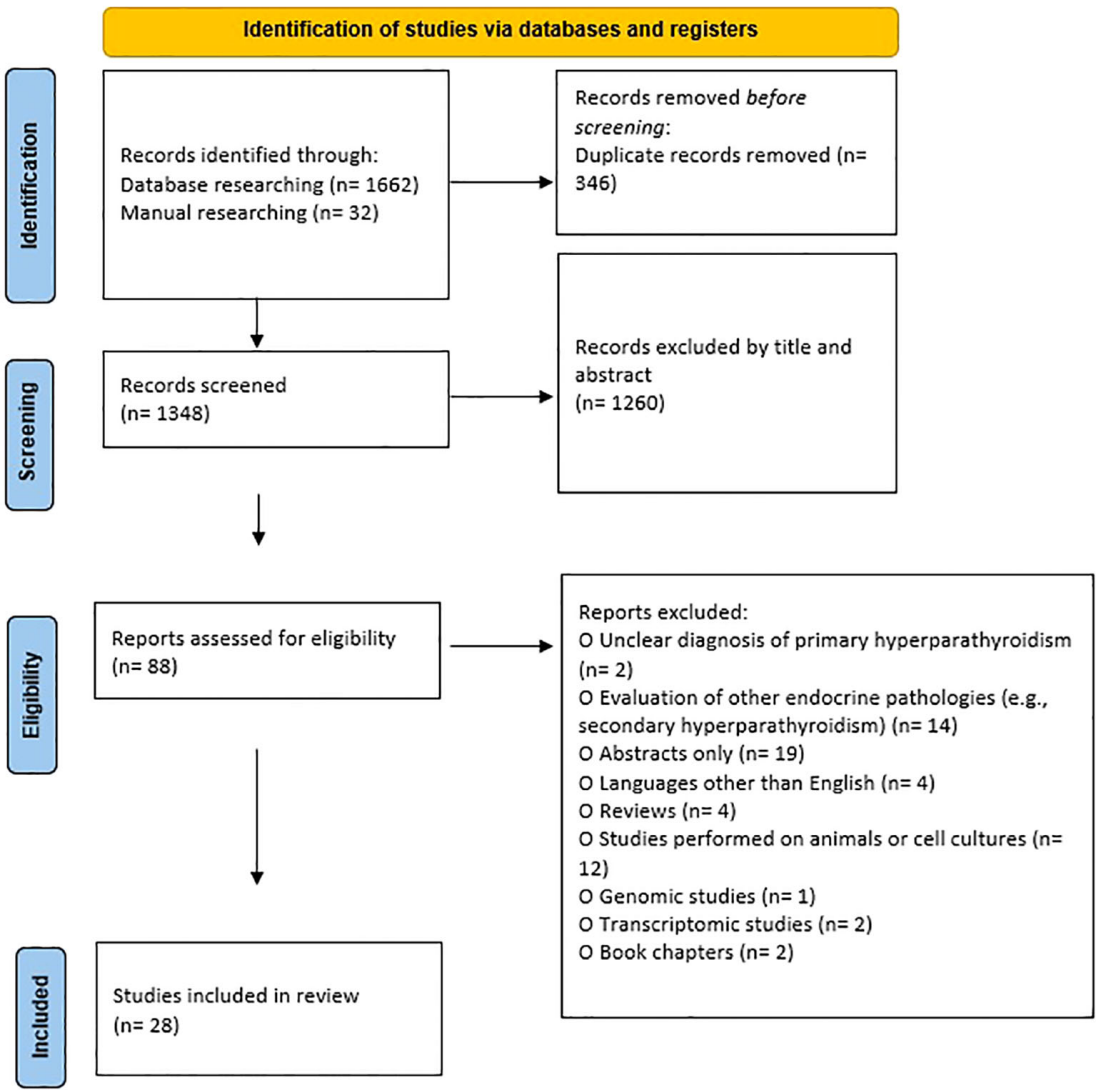
PRISMA style flowchart of the selected studies.

The screening of literature was performed by two researchers (AGM and OSP) independently, taking into consideration several inclusion and exclusion criteria. The following inclusion criteria were used: 1) clear diagnosis of PHPT, 2) studies evaluating markers of chronic inflammation, 3) studies in English, and 4) studies on human subjects. The exclusion criteria were: 1) unclear diagnosis of PHPT, 2) evaluation of other endocrine pathologies (e.g. secondary hyperparathyroidism), 3) abstracts only, 4) languages other than English, 5) reviews, 6) studies performed on animals or cell cultures, 7) book chapters, 8) genomic studies, 9) transcriptomic studies, and 10) studies performed on a duplicate group of patients. At the same time, the two researchers extracted data and evaluated the quality of the recruited studies. Any discrepancy was resolved after consultation with the third investigator (GC). Additionally, a manual search was performed in Google Scholar and in the references of found articles and reviews to identify articles that might have been missed. For each article, the extracted information included: the first author’s name, year of publication, country, sample size, available characteristics of the groups (age, gender, BMI), type of biological sample used, evaluated markers and outcomes. The literature search was carried out in line with the PRISMA guidelines (**Figure 1**) (22).

The studies were assessed for methodological quality using the Newcastle Ottawa Scale (NOS) recommended for systematic reviews of non-randomized studies. The original Newcastle Ottawa Scales for case-control and cohort studies (23) and an adapted version for cross- sectional studies (24) were used to address the risk of bias (ROB) of the included studies. The evaluation focused specifically on selection, comparability and outcome for the cross- sectional and cohort studies and selection, comparability and exposure for case- control studies. Articles were graded using a star allocation scheme. Eight items (9 possible stars*) were assessed for the case- control and cohort studies and seven items (10 possible stars*) were analyzed for the cross-sectional studies. Articles with 8 or more stars were categorized as low ROB, those with 6 to 7 stars as medium ROB and those with 5 or less stars allocated were classified as high ROB (23, 24).

## Results

### Literature research

Following the electronic database search, 1662 articles were identified. Additionally, 32 articles were retrieved by hand searching. After exclusion of the duplicates (n=346), 1348 articles remained. Subsequently, after evaluating the title and abstract, 1260 irrelevant articles were excluded. The remaining studies were assessed for eligibility: two articles were excluded because the criteria for PHPT diagnosis were not clearly stated, fourteen articles evaluating other endocrine diseases were also excluded and nineteen abstracts without full-text articles or for whom full-text articles could not be retrieved were excluded as well. Additionally, four studies written in languages other than English, twelve articles containing studies performed on animals or cell cultures, one genomic and two transcriptomic studies were also excluded, along with four reviews and two book chapters. Twenty-eight articles were included in the review. In total, 1572 patients with PHPT were evaluated. The characteristics of the groups evaluated in the studies, along with the markers and outcomes, are summarized in **Table 1**.

**Table 1:** Markers of chronic inflammation in primary hyperparathyroidism.

Authors (year) and reference	Country	Study type	Study population characteristics	Biological sample	Markers and outcomes	NOS	ROB
Beysel et al. (2019) (25)	Turkey	Longitudinal, retrospective Case- control	° PHPT group (n= 55; age= 51.85 ± 10.21 y; 78.2% female, BMI= 30.58 ± 5.14 kg/m^2^) °Control group (n= 50; age= 53.31 ± 7.84 y; 72% female; BMI= 28.55 ± 3.71 kg/m^2^)	Blood serum	*Baseline:* PLR, RDW, Hs-CRP: ↑ in the PHPT group LC: ↓ in the PHPT group WBC: similar in PHPT patients and controls *6 months after PTX:* Hs-CRP, PLR, RDW: ↑ in PHPT group vs control group Hs-CRP, LC, PLR, RDW: similar compared to preoperative values	6	M
Lam et al. (2018) (26)	Taiwan	Longitudinal, retrospective Cohort	° PHPT (n= 95; age= 59 (51 – 67) y; 76% female): - Asymptomatic: n= 52; age= 62 (56 – 68) y; 85% female, BMI= 23.7 (21.3 – 26.3) kg/m^2^ - Symptomatic: n= 43; age= 56 (48 – 62) y; 65% female, BMI= 23.7 (22.0 – 27.1) kg/m^2^ -*Follow- up group* (n= 30)	Blood	*6 months after PTX:* NLR: ↓ compared to preoperative values WBC: similar compared to preoperative values	7	M
Chertok-Shacham et al. (2008) (27)	Israel	Cross- sectional	° PHPT group (n= 35; age= 57.5 ± 10.8 y; 88% female; BMI= 27.8 ± 6.0 kg/m^2^): - Severe PTH (n= 15) - Mild PHPT (n= 20) °Control group (n= 25; age= 59.7 ± 7.9 y; 84% female; BMI= 29.8 ± 5.4 kg/m^2^)	Blood serum	Fibrinogen: ↑ in mild PHPT and controls *vs.* severe PHPT CRP, IL-6, WBC: similar in PHPT patients and controls	7	M
Grey et al. (1996) (28)	USA	Cross- sectional	° PHPT group (n= 38; age= 65 ± 3 y; 78% female): -*Follow- up group* (n= 7) ° hPT group (n= 6; age= 54 ± 9 y; 83% female) °Control group (n= 12; age= 58 ± 2 y; 83% female)	Blood serum	*Baseline:* TNF-α, IL-6, sIL-6R: ↑ in PHPT patients *vs* controls IL-1β: similar in PHPT patients and controls *After PTX:* TNF-α, IL-6, sIL-6R: ↓	7	M
Nakchbandi et al. (2002) (29)	USA	Longitudinal, prospective Cohort	° PHPT (n= 29; age= 63 ± 2 y; 89% female): *-Follow- up group* (n= 26)	Blood serum	*Baseline:* IL-6, sIL-6R: ↑ in the study group *Follow-up for 22 months:* IL-6: stable sIL-6R: slight ↑	8	L
Alakuş et al. (2021) (30)	Turkey	Longitudinal, retrospective Cohort	° PHPT (n= 37; median age= 54 y; 89.2% female)	Blood	*6 months after PTX:* NLR: ↓ compared to preoperative values WBC: similar compared to preoperative values	8	L
Halabe et al. (2000) (31)	Israel	Longitudinal Cohort	° PHPT (n= 10; age range= 45–78 y; 50% female)	Blood (serum+ tissue culture supernatants of PHA-stimulated lymphocytes)	*Baseline:* IL-2R: normal values (serum+ supernatant) IL-6 in supernatant: ↑ *3 weeks after PTX:* Serum IL-2R: ↑ IL-6, IL-2R in supernatant: ↑	6	M
Shasha et al. (1989) (32)	Israel	Longitudinal Case- control	° PHPT (n= 3, aged 25 y, 64 y and 66 y, respectively; 66% female) °Control group (n= 3, aged 27 y, 60 y and 62 y, respectively; 66% female) ° Lipoma of the neck (n= 1; age= 58 y; male)	Blood	*Baseline:* S: ↑ in PHPT patients *vs* controls TTC, H/S: ↓ in PHPT patients *vs* controls LSLT: inhibited *1 month after surgery:* TTC, H/S: ↑ S: ↓ LSLT: restored to normal values	6	M
Bollerslev et al. (2009) (33)	Multicentric (Sweden, Norway, Denmark)	Cross- sectional	° Mild PHPT (n= 116; age= 63 ± 8 y; 83% female): - Observation for 2 years (n= 62; age= 63 ± 7 y; 85% female; BMI= 27 ± 4 kg/m^2^) - Surgery (n= 54; age= 63 ± 8 y; 81% female; BMI= 27 ± 4 kg/m^2^)	Blood serum	Hs-CRP: similar between groups (observation vs surgery), no change in the observation period IL-1Rα: no change in the observation period; not influenced by PTX	10	L
Kotzmann et al. (1998) (34)	Austria	Longitudinal Case- control	° PHPT group (n= 12; mean age= 56.6 y; 75% female) °Control group (n= 9; 66% female)	Blood (serum+ lymphocytes+ peripheral blood mononuclear cells)	*Baseline:* Ig A, Ig M, Ig G: similar in PHPT patients and controls H: ↑ in PHPT group *vs* controls S: ↓ in PHPT group *vs* controls H/S: ↑ in PHPT group *vs* controls LPRP: suppressed in PHPT group *6 months after PTX:* Ig A, Ig M, Ig G, H, S: similar compared to preoperative values LPRP: restored to normal values	7	M
Alay et al. (2020) (35)	Turkey	Longitudinal, prospective Case- control	° PHPT group (n= 28; age= 57.7 ± 10.9 y; 100% female; BMI= 26.41 ± 4.18 kg/m^2^) °Control group (n= 27; age= 53.3 ± 9.31 y; 100% female; BMI= 28.6 ± 5.22 kg/m^2^)	Blood serum	Fibrinogen: ↑ in PHPT patients *vs* controls	6	M
Nakchbandi et al. (2001) (36)	USA	Longitudinal Case- control	° PHPT group (n= 29): -*Follow-up* group (n= 3) ° hPT group (n= 7) °Control group (n= 22)	Blood serum	*Baseline:* IL-6: ↑ in PHPT patients *vs* controls IL-11: ↓ in PHPT patients *vs* controls and hPT patients *After PTX (evaluated every 4 h for 24 h):* IL-6: ↓ IL-11: ↑	5	H
Guo et al. (2000) (37)	UK	Longitudinal Case- control	° PHPT group (n= 8; age= 55 ± 11 y; 100% female) ° Healthy controls (n= 8) ° Surgical controls (n= 5)	Blood serum	*Baseline:* IL-6: ↑ in PHPT group *vs* controls *After PTX (at 1h, 2 h, and 1, 2, 5, 7 and 12 days):* IL-6: ↑ immediately (after 1–2 h) with a maximum increase 1 day after PTX	7	M
Almqvist et al. (2011) (38)	Sweden	Longitudinal Cohort	° PHPT group (n= 45; age= 69.7 ± 8.4 y; 91% female)	Blood serum/plasma	*Baseline:* Hs-CRP: 18% of patients had values above the upper limit of reference range IL-6: 7% had values above the upper limit of reference range ESR: 24% had values above the upper limit of reference range *1 year after PTX:* Hs-CRP, IL-6, ESR: ↑	8	L
Ogard et al. (2005) (39)	Denmark	Longitudinal Case- control	° PHPT group (n= 45; age= 59.5 ± 9.4 y; 82% female; BMI= 25.7 ± 3.7 kg/m^2^): -*Follow-up group* (n= 17) °Control group (n= 40; age= 58.1 ± 11.1 y; 82.5% female; BMI= 24.7 ± 3.9 kg/m^2^)	Blood plasma	*Baseline*: Hs-CRP, TNF-α: ↑ in the PHPT group *vs* controls IL-6: similar in PHPT group and controls *After PTX (at 7 and 18 months):* Hs-CRP, TNF-α, IL-6: similar compared to preoperative values	8	L
Emam et al. (2012) (40)	Egypt	Cross- sectional	° Asymptomatic PHPT group (n= 26; age= 52.8 ± 4.4 y; 65% female) °Control group (n= 22; age= 51.7 ± 5.9 y; 68% female)	Blood serum	IL-6, Hs-CRP: ↑ in the asymptomatic PHPT group *vs* controls	8	L
Verheyen et al. (2017) (41)	Austria	Cross- sectional	° PHPT group (n= 136; age= 67 ± 10 y; 79% female; BMI= 28 ± 5 kg/m^2^)	Blood serum	CRP	5	H
Christensen et al. (2015) (42)	Norway	Longitudinal, prospective Case- control	° PHPT (n= 57; mean age= 59.7 y; 84.2% female; BMI= 26 kg/m^2^) °Control group (n= 20; mean age= 56.8 y; 75% female; BMI= 25.3 kg/m^2^)	Blood serum/plasma	*Baseline:* MMP9, S100A4 protein, sCD14: ↑ in the PHPT group *vs* controls S100A8/A9, RAGE: similar in PHPT patients and controls *After PTX (at 1, 3 and 6 months):* CRP, MMP9: ↑ sCD14, S100A4: ↓ RAGE: ↑ 1 month after PTX, but ↓ thereafter	6	M
Zeren et al. (2015) (43)	Turkey	Longitudinal, retrospective Cohort	° PHPT (n= 32; age= 53.2 ± 12.5 y; 75% female) - adenoma (n= 29) - carcinoma (n= 3)	Blood	NLR	7	M
Kahal et al. (2012) (44)	UK	Longitudinal, prospective Case- control	° PHPT group (n= 24; age= 60 ± 13.6 y; 93.1% female) ° HT group (n= 23; age= 43 ± 14.6 y; 70.4% female)	Blood serum	*Baseline:* CRP: similar between groups *3 months after PTX:* CRP: similar to preoperative values	6	M
Harmantepe et al. (2024) (45)	Turkey	Longitudinal, retrospective Case- control	° PHPT (n= 203; age= 54.08 ± 11.0 y; 86.6% female) °Control group (n= 97; age= 56.74 ± 14.02 y; 85.8% female)	Blood	*Baseline:* NLR, PLR, SII: ↓ in the PHPT group *vs* controls LMR: similar between groups *6 months after PTX:* NLR, SII: ↑ compared to preoperative values LMR: ↓ compared to preoperative values PLR: similar to preoperative values NLR, PLR, SII, LMR: similar compared to control group	6	M
Christensen et al. (2012) (46)	Norway	Longitudinal Case- control	° PHPT (n= 57; age= 59.7 ± 12.2 y; 84% female; BMI= 26.0 ± 4.35 kg/m^2^) °Control group (n= 20; age= 56.8 ± 4.34 y; 75% female, BMI= 25.3 ± 2.97 kg/m^2^)	Blood plasma	*Baseline:* KTR, kynurenine: ↑ in PHPT group *vs* controls Tryptophan, anthranilic acid, xanthurenic acid: ↓ in PHPT group *vs* controls Neopterin, CRP: similar between groups *6 months after PTX:* Anthranilic acid, CRP: ↑ Neopterin: ↓ KTR, kynurenine, tryptophan, xanthurenic acid: similar to preoperative values	7	M
Yankova et al. (2023) (47)	Bulgaria	Cross-sectional	° PHPT only (n= 50; age= 58.3 ± 8.7 y; 94% female; BMI= 26.7 (23.3 - 30.1) kg/m^2^) ° PHPT and HaT (n= 50; age= 62.9 ± 8.3 y; 96% female; BMI= 26.7 (23.9 - 31.3) kg/m^2^) ° Healthy controls (n= 37; age= 58.4 ± 6.5 y; 94.6% female, BMI= 28.7 ± 6.3 kg/m^2^) °Control group with HaT (n= 37; age= 60.1 ± 7.5 y; 94.6% female, BMI= 28.6 ± 6.1 kg/m^2^)	Blood serum	IL-17A: ↑ in PHPT groups *vs* controls, but similar in PHPT patients with HaT *vs* PHPT patients without HaT	8	L
Dozio et al. (2020) (48)	Italy	Cross-sectional	° PHPT (n= 50; age= 66.5 (58.8 - 74.0) y; 100% female; BMI= 25.6 (22.9 - 28.7) kg/m^2^) °Control group (n= 20; age= 69.0 (57.0 - 75.0) y; 100% female, BMI= 25.1 (23.0 - 28.6) kg/m^2^)	Blood plasma	IL-17A: similar in PHPT group *vs* controls	8	L
Patel et al. (2014) (49)	USA	Longitudinal, Cohort	° PHPT (n= 43; age= 56.2 ± 12.1 y; 77% female; BMI= 29.4 ± 5.5 kg/m^2^)	Blood serum	*After PTX:* MCP-1: ↓ compared to preoperative values	6	M
Meng et al. (2025) (50)	USA	Longitudinal, prospective Case- control	° PHPT group (n= 70; age= 61 ± 12 y; 77% female; BMI= 29.9 ± 6.4 kg/m^2^): -*Follow-up group* (n= 28, age= 61 ± 11 y; 75% female) °Control group (n= 70; age= 60 ± 8 y; 80% female; BMI= 28.9 ± 2.7 kg/m^2^)	Blood serum	*Baseline*: CRP, IL-6, MCP-1: ↑ in the PHPT group *vs* controls IL-10: similar in PHPT group and controls *3 months after PTX:* MCP-1, IL-6: ↓ compared to preoperative values CRP, IL-10: similar to preoperative values	8	L
Deniz et al. (2024) (51)	Turkey	Longitudinal, retrospective Cohort	° PHPT (n= 70; age= 50.45 ± 12.3 y; 84% female)	Blood	*After PTX:* MHR: ↑ compared to preoperative values SII, PLR: ↓ compared to preoperative values SIRI: similar to preoperative values	7	M
Bulbul et al. (2025) (52)	Turkey	Longitudinal, retrospective Cohort	° PHPT (n= 119; age= 53 ± 14 y; 78.2% female)	Blood	*1 month after PTX:* PLR: ↑ compared to preoperative values NLR, MHR: ↓ compared to preoperative values	6	M

NOS, Newcastle-Ottawa Score; ROB, risk of bias; M, medium; L, low; H, high; y, years; kg, kilograms; PTX, parathyroidectomy; HT, hemithyroidectomy; LC, lymphocyte count; WBC, white blood cell count; RDW, red blood cell distribution width; PLR, platelet to lymphocyte ratio; LMR, lymphocyte to monocyte ratio; Hs-CRP, High sensitivity C-Reactive Protein; NLR, neutrophil to lymphocyte ratio; CRP, C-Reactive Protein; hPT, hypoparathyroidism; IL-6, interleukin-6; sIL-6R, soluble interleukin-6 receptor; IL-1β, interleukin 1 beta; TNF-α, tumor necrosis factor alpha; IL-2R, receptor for interleukin-2; PHA, phytohemagglutinin; TTC, total amount of T cells; S, absolute count of CD8+ suppressor T cells; H/S, helper to suppressor ratio; LSLT, lectin-stimulated lymphocyte transformation; IL-1Rα, alpha subunit of IL-1 receptor; H, absolute count of CD4+ helper T cells; LPRP, lymphocyte proliferation response to phytohemagglutinin; Ig, immunoglobulin; ESR, erythrocyte sedimentation rate; MMP9, matrix metallopeptidase 9; sCD14, soluble CD14; RAGE, receptor for advanced glycation end products; SII, systemic immune inflammation index; KTR, kynurenine-to-tryptophan ratio; BTD, Benign thyroid disease; HaT, Hashimoto thyroiditis; MCP-1, monocyte chemoattractant protein-1; MHR, monocyte-to-high-density lipoprotein cholesterol ratio; SIRI, systemic inflammation response index.

### Study quality assessment

The assessment of methodological quality for the reviewed studies demonstrated that 17 articles (60.7%) had a medium risk of bias, 9 (32.1%) had a low risk of bias, and the remaining 2 (7.2%) exhibited a high risk of bias according to NOS.

### Cytokines and C-Reactive protein

In the study performed by Grey et al. (28), the mean level of IL-6 in untreated PHPT patients (18.6 ± 2.1 pg/ml) was higher compared with controls (1.1 ± 0.1 pg/ml). Among the seven individuals who underwent successful surgical treatment, IL-6 levels showed a significant reduction, from a mean value of 6.8 ± 2.1 pg/ml preoperatively to 0.6 ± 0.1 pg/ml postoperatively (p = 0.01). Likewise, IL-6 soluble receptor (sIL-6R) and TNF-α values were significantly higher compared with controls (p < 0.001), but IL-1β level was similar in the two groups. Furthermore, after parathyroidectomy, both sIL-6R and TNF-α decreased into the normal range. In PHPT patients, a significant correlation was identified between PTH and IL-6 (r = 0.47, p = 0.003)/TNF-α (r = 0.41, p = 0.01). Additionally, IL-6 was found to be strongly correlated with several markers of bone resorption: serum deoxypyridinoline (s-DPD) and type 1 collagen carboxyterminal telopeptide (s-ICTP), urinary pyridinoline (u-PD) and deoxypyridinoline (u-DPD). TNF-α was also correlated with these markers, but the significance was lost after controlling for IL-6. IL-6 levels were found to be positively correlated with osteocalcin (OC) levels (r = 0.57, p < 0.001), but not with alkaline phosphatase (AP) levels (28).

In a study with a similar design (29), elevated levels of IL-6 and sIL-6R in PHPT patients, compared with controls, were confirmed. Baseline and mean IL-6 levels for each patient showed a significant correlation with baseline and mean PTH values (r = 0.44, p < 0.05). Relationships with bone markers were also assessed revealing that baseline IL-6 correlated with s-ICTP (r = 0.54, p < 0.005), IL-6sR showed a correlation with serum N-telopeptide of type I collagen (r = 0.50, p < 0.005), both markers were associated with u-DPD and neither with OC. Baseline values of sIL-6R correlated significantly with the annual decline in bone mineral density at the total femur (r = - 0.53, p < 0.01). Moreover, the authors found that serum sIL-6R and IL-6 cutoffs of ≥ 45.6 ng/ml and ≥ 11.8 pg/ml, respectively, identified subjects with significantly greater rates of bone loss at the total femur within the study group (29).

Halabe et al. (31) showed that in patients with PHPT, levels of the receptor for interleukin-2 (IL-2R), both in the serum and in tissue culture supernatant, were normal prior to parathyroidectomy and significantly increased after surgery (p < 0.05). However, IL-2R levels were elevated in PHPT patients compared with healthy donors. IL-6 production in tissue culture supernatants from phytohemagglutinin (PHA)-stimulated lymphocytes was higher preoperatively and increased further after surgery (p < 0.005). In comparison with healthy controls, IL-6 production was also greater in PHPT patients (31).

Mean levels of circulating IL-11 were lower in PHPT patients compared with controls (5.7 ± 1.2 pg/ml vs 12.4 ± 1.0 pg/ml, p < 0.001) in a study by Nakchbandi et al. (36) A strong negative correlation between circulating PTH and serum IL-11 (r = - 0.61, p < 0.001) was also revealed. Levels of IL-6 and IL-11 were measured every 4 hours for the first 24 hours after surgery in three patients who underwent successful parathyroidectomy. A rapid decline in the mean value of IL-6 was identified (21.5 ± 2.7 pg/ml preoperatively vs 7.1 ± 0.6 pg/ml 24 h postoperatively, p = 0.03), together with a rise in IL-11 levels (4.3 ± 0.7 pg/ml preoperatively vs 9.9 ± 1.7 pg/ml 24 h postoperatively, p = 0.03) (36).

Ogard et al. (39) found that plasma TNF-α levels were significantly higher in the PHPT group compared with controls (p < 0.01), while IL-6 levels did not show a significant difference between groups (p = 0.17). Additionally, plasma CRP levels were higher in PHPT patients than in controls (p = 0.002). Notably, ten PHPT patients had CRP levels exceeding 3 mg/l, whereas none of the individuals in the control group had CRP values above this threshold. A correlation between log CRP and log IL-6 was also identified (r = 0.59, p < 0.001), but no significant correlations were found between log IL-6 and PTH or between any of the evaluated inflammatory markers and age, bone mineral density (BMD), T-score or Z-score. A small group of only 17 patients was assessed at 7 and 18 months after parathyroidectomy, and no significant differences between the pre-and postoperative values of CRP, TNF-α and IL-6 were observed (39). Likewise, in the study by Chertok-Shacham et al., levels of IL-6 in PHPT patients were similar to those in controls, but they showed a significant correlation with both calcium (p = 0.046) and PTH (p = 0.029). Also, CRP levels did not differ significantly between PHPT patients and controls, but a positive correlation between calcium and CRP (p = 0.037) was identified (27). In contrast, levels of IL-6 were significantly elevated in PHPT patients compared with healthy controls (p = 0.001) in another research (37) and increased after parathyroidectomy, with a maximum level one day after surgery. However, a rise in IL-6 levels was also noticed in surgical controls, with a peak value observed two days after surgery (37). Meng et al. (50) also identified higher values of IL-6 in patients with PHPT compared with controls (p < 0.001) and a significant positive correlation between PTH and this cytokine (r = 0.31, p < 0.05). Although IL-6 levels decreased at the 3-month follow-up visit after parathyroidectomy (p < 0.05), they remained significantly higher than those observed in control patients (p < 0.01).

Almqvist et al. evaluated a cohort of PHPT patients and found that some displayed inflammatory markers above the upper reference limit. Notably, the mean values of IL-6 (p < 0.001), ESR (p < 0.001) and CRP (p < 0.01), were higher one year after parathyroidectomy, compared with preoperative levels. Subgroup analysis revealed that the increases in IL-6 and ESR were statistically significant in both parathyroid adenoma and multiglandular disease cases (38).

No significant changes in CRP concentrations after parathyroidectomy were observed in three other studies (25, 44, 49), despite higher Hs-CRP in PHPT patients compared with controls (p < 0.05), both pre- and postoperatively, and a positive correlation between PTH and Hs-CRP preoperatively (r = 0.377, p = 0.001) being identified in one of the studies (25). Meng et al. (50) reported higher preoperative CRP levels in PHPT patients compared with controls (p = 0.02) as well, but similar post-parathyroidectomy values between the two groups.

The Scandinavian Study on Primary Hyperparathyroidism (SIPH) enrolled 116 patients randomized into two groups (surgery and observation) and followed-up for two years. The authors found no significant changes in Hs-CRP during this observation period and no differences between the two groups regarding this parameter (33).

In a research confined to asymptomatic PHPT, levels of Hs-CRP and IL-6 were significantly higher compared with controls (p < 0.001) and appeared to be strongly correlated with PTH levels (Hs-CRP: r = 0.82, p < 0.001 and IL-6: r = 0.787, p < 0.001, respectively) (40).

Using cross-sectional data from the Eplerenone in Primary Hyperparathyroidism (EPATH) Study, Verheyen et al. found mean baseline CRP values of 0.15 (0.6 – 2.7) mg/l in patients with PHPT and showed that PTH was not significantly correlated with CRP (41).

Two studies assessed IL-17A levels in patients with PHPT (47, 48). Dozio et al. (48) found similar circulating IL-17A levels in osteoporotic postmenopausal women with and without PHPT, whereas Yankova et al. (47) reported significantly elevated serum levels of IL-17A in PHPT patients compared with controls (p < 0.001), and no differences between PHPT patients with and without Hashimoto’s thyroiditis (HaT). Both studies observed no significant differences in receptor activator of NF-*κ*B ligand (RANKL), osteoprotegerin (OPG), or the RANKL/OPG ratio between PHPT and control groups. In PHPT patients, Dozio et al. (48) identified significant negative correlations between IL-17A and ionized calcium (r = –0.294, p = 0.047) and urinary calcium excretion (r = –0.300, p = 0.045), and positive correlations with T-scores at the femoral neck (r = 0.364, p = 0.021) and total hip (r = 0.353, p = 0.015). Yankova et al. (47) found positive correlations between IL-17A and calcium (r = 0.33, p < 0.001), PTH (r = 0.27, p < 0.001), and RANKL (r = 0.167, p = 0.03), and negative correlations with phosphate (r = –0.29, p < 0.001), 25 OH vitamin D (r = –0.26, p = 0.001), and OC (r = –0.15, p = 0.05); these findings remained unchanged after controlling for HaT.

Meng et al. (50) assessed circulating IL-10 levels and found no significant differences between PHPT patients and controls, both pre- and postoperatively. Furthermore, no significant changes in IL-10 levels were observed following parathyroidectomy.

### Full blood count derived parameters

In a cohort of 203 PTHP patients, Harmantepe et al. evaluated the following indices derived from the FBC: NLR, PLR, LMR and SII. PLR (p = 0.01), NLR (p < 0.05), and SII (p < 0.05) values were significantly decreased in PHPT cases compared with controls, while no significant difference was found for LMR (p = 0.19). Six months after parathyroidectomy, NLR and SII significantly increased (p = 0.026 and p = 0.016, respectively), while LMR showed a significant decrease (p = 0.023). Despite these changes, no statistically significant differences were observed between PHPT patients and controls for any of the four parameters (45).

In the study by Beysel et al., no significant differences were observed in platelet count and WBC between PHPT cases and controls. However, the lymphocyte count was significantly lower in the PHPT group (p < 0.05). PLR and RDW were significantly higher in PHPT patients, both before and after surgery, when compared with controls (p < 0.05), but no significant changes in PLR and RDW values were identified following surgery in PHPT patients. Additionally, PTH concentration was found to be positively correlated with preoperative PLR (r² = 0.234, p = 0.023) and RDW (r² = 0.296, p = 0.004) (25).

In a cohort of 95 PHPT patients (26), median values of 7000/μl (interquartile range- IQR= 5700–8500) for the WBC and 2.20 (IQR= 1.66– 2.81) for NLR were described and both parameters were positively associated with serum calcium (p = 0.001) and PTH (p = 0.013). Moreover, the authors revealed that NLR had lower values in patients with more than one parathyroid gland affected compared with those with single gland disease (p = 0.054). The postoperative NLR was significantly lower in patients cured of PHPT compared with preoperative values, but the WBC and platelet count did not differ significantly (26).

Likewise, the NLR was significantly higher preoperatively in another study (30) and reported to be correlated positively with PTH [r = 0.519, p = 0.001 in (30) and r = 0.472, p = 0.006 in (43)], serum calcium [r = 0.390, p = 0.017 in (30) and r = 0.513, p = 0.003 in (43)] and WBC (r = 0.531, p = 0.001 in (30)) and negatively with serum phosphate (r = -0.331, p = 0.046 in (30)). Zeren et al. also discovered a significant positive association between this marker and the diameter of parathyroid adenomas (r = 0.675, p < 0.001), or the presence of parathyroid carcinoma (r = 0.578, p = 0.001) (43).

The effects of parathyroidectomy on FBC- derived markers were assessed in two additional retrospective cohort studies. Deniz et al. (51) demonstrated that SII and PLR significantly decreased (p = 0.0001) during the follow-up period, which ranged from 7 months to 2 years, whereas MHR (p = 0.0001) increased, and SIRI remained unchanged relative to preoperative values. Moreover, RDW increased and NLR decreased, although these changes did not achieve statistical significance. In the study by Bulbul et al. (52), one month after parathyroidectomy, PLR (p = 0.024) was higher, while NLR (p = 0.011) and MHR (p = 0.019) were lower compared with preoperative values. A significant positive correlation was found between postoperative PTH values and NLR (r = 0.227, p = 0.046) (52).

The T- lymphocytes pattern was assessed in three patients with PHPT compared with controls and a lower count of total T lymphocytes (TTC), a higher count of T suppressor cells and a significantly lower helper to suppressor ratio (H/S) were identified (32). The authors also noted that the lectin-stimulated lymphocyte transformation (LSLT) was significantly inhibited in PHPT patients. Parathyroidectomy resulted in an increase of 10-60% in TTC in each patient, an increase in T helper cells (26%) and a significant decrease (36.4%) in T suppressor cells, leading to a normalization of the H/S ratio. The LSLT increased significantly after parathyroidectomy from a mean of 12.357 ± 7.28 to a mean of 54.803 ± 25.246 cpm (p < 0.01) when PHA was used and from 8.772 ± 5.276 to 25.403 ± 7.914 (p < 0.05) when concanavalin A was used (32).

In the study by Kotzmann et al. (34), the phenotype and function of mononuclear cells in PHPT patients were analyzed. The serum immunoglobulin levels (IgG, IgM, IgA) were found to be within normal range, with no significant differences observed between patients with PHPT (both pre- and post-operatively) and controls. The authors noted significantly elevated levels of T helper cells and decreased T suppressor cells preoperatively and subsequently a high H/S ratio in PHPT patients compared with controls. The levels of these markers were not significantly influenced by surgery. Peripheral blood lymphocytes obtained from patients with PHPT postoperatively were incubated with autologous serum obtained preoperatively and serum from healthy controls, respectively. The lymphocyte proliferation response to PHA in the highest concentration tested was significantly reduced when autologous serum was used compared with the response obtained when serum from normal control subjects was used. Normalization of the proliferation response was observed when the same cells were incubated with serum obtained post-operatively (34).

### Other inflammatory markers

Christensen et al. evaluated tryptophan (T) metabolites and the kynurenine to tryptophan ratio (K/T), as systemic markers of Interferon (IFN)γ mediated immune activation, and found that levels of kynurenine (p = 0.029) and the K/T ratio (p= 0.015) were significantly higher in PHPT patients, while T (p = 0.007), anthranilic acid (p = 0.013) and xanthurenic acid (p = 0.013) were significantly lower compared with controls. There were no differences between the two groups regarding CRP. Levels of plasma B6 vitamers pyridoxal 5-phosphate (PLP) (p = 0.007) and pyridoxal (p = 0.013) were significantly lower in PHPT patients. PTH was negatively correlated with PLP (r = - 0.27, p= 0.048) and with CRP (r = - 0.313, p = 0.024). Patients were reevaluated at 1, 3 and 6 months after surgery. There was a significant increase in the levels of PLP, anthranilic acid and CRP after 6 months of follow-up (46).

Higher fibrinogen levels were detected in patients with mild PHPT and controls compared with those exhibiting severe PHPT by Chertok-Shacham et al. (27), whereas Alay et al. found that fibrinogen levels were significantly elevated in PHPT patients compared with controls (338.78 ± 63.87 mg/dL vs 304.30 ± 45.67 mg/dL, p = 0.041), and identified a significant positive correlation between the volume of parathyroid adenomas and this inflammatory marker in the PHPT group (r = 0.711, p = 0.001) (35).

Two studies (49, 50) evaluated monocyte chemoattractant protein-1 (MCP-1). Meng et al. (50) found significantly higher levels in patients with PHPT compared with controls (p < 0.001), which decreased three months post-parathyroidectomy (p < 0.05), reaching values comparable to those of controls. Significantly lower levels of MCP-1 were also observed 15 to 20 minutes after parathyroidectomy by Patel et al. (p < 0.001). Additionally, positive correlations between PTH and MCP-1 were identified in both studies: r = 0.47, p < 0.01 in (49) and r = 0.33, p < 0.05 in (50).

Christensen et al. compared CRP and several emerging inflammatory markers between PHPT patients and healthy blood donors, revealing that the levels of serum matrix metalloproteinase-9 (MMP9) (p = 0.029), serum soluble CD14- sCD14 (p = 0.002) and plasma protein S100A4 (p < 0.001) were significantly higher in PHPT patients. However, there were no significant differences between the two groups for CRP (p = 0.374), plasma protein S100A8/A9 (p = 0.157), and serum receptor for advanced glycation end products (RAGE) (p = 0.889). None of the inflammatory markers evaluated were correlated with PTH or ionized calcium. Inflammatory markers were reevaluated at 1, 3 and 6 months after surgery. MMP9 increased, this change reaching statistical significance at 6 months after surgery (p = 0.017), while S100A4 and sCD14 decreased during follow-up. A significant decrease in sCD14 (p = 0.045) was observed after 1 month, while S100A4 values were significantly changed throughout the entire follow-up period (p = 0.022). RAGE increased significantly one month after parathyroidectomy (p = 0.001) but decreased afterwards (42).

## Discussion

The present review, conducted in a systematic manner, identified a series of markers associated with chronic inflammation that are altered in PHPT patients. In most studies, pro-inflammatory markers such as TNF-α, IL-6, Hs-CRP, and fibrinogen were found to be elevated in patients with PHPT. While WBC values were rather similar in patients and controls, for other markers derived from the FBC (NLR, PLR or RDW) significant differences were found. Data on the impact of parathyroidectomy on inflammation parameters were conflicting.

**Figure 2:**
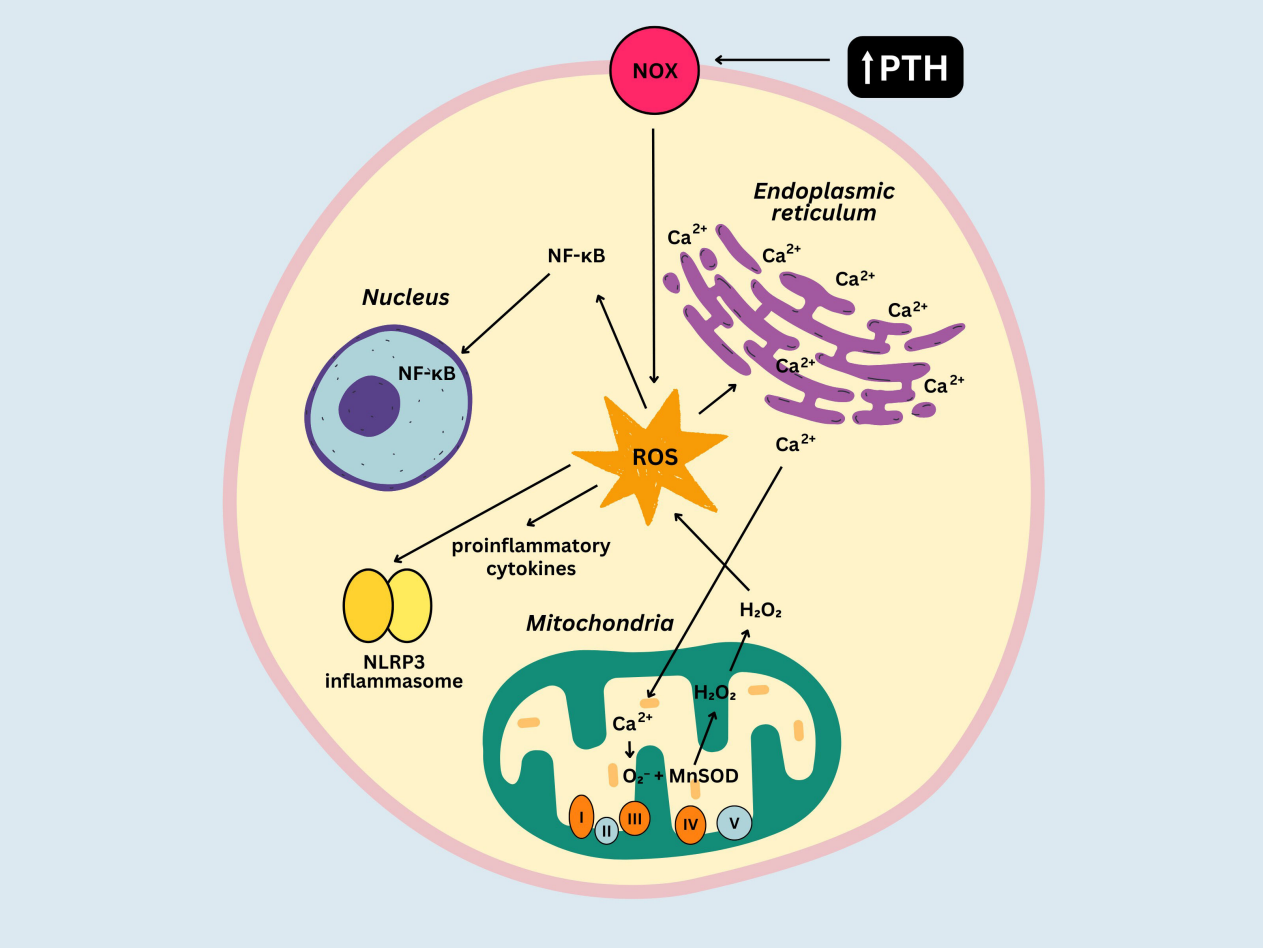
Relationship between oxidative stress and inflammation in primary hyperparathyroidism. NOX, NADPH oxidase; ROS, reactive oxygen species; MnSOD, manganese superoxide dismutase; NF-κb, nuclear factor-kappa B; O_2_^-^, superoxide anion.

Several mechanisms might be responsible for the development of chronic inflammation in PHPT. Calcium is essential for various physiological processes, including muscle contraction, cell signaling and bone homeostasis, but plays a crucial role in regulating the immune response as well. For example, it contributes to the activation and differentiation of immune cells and the synthesis of inflammatory mediators (53). Possible ways in which excessive amounts of calcium can promote chronic inflammation include: 1) overstimulation of immune cells that are normally activated by signaling pathways involving calcium (macrophages, T cells), 2) overproduction of pro-inflammatory cytokines (IL-1, IL-6, and TNF-α) (54, 55) and 3) metabolism enhancement (mitochondrial calcium stimulates the tricarboxylic acid cycle and oxidative phosphorylation), leading to electron leakage from the electron transport chain (ETC) and reactive oxygen species (ROS) production (56). Superoxide anion (O_2_^-^) is a highly reactive ROS that is generated as a result of electron leakage from the ETC complexes (particularly Complex I and III) and reacts with manganese superoxide dismutase (MnSOD), an antioxidant enzyme located in the mitochondrial matrix, forming hydrogen peroxide (H_2_O_2_). H_2_O_2_ is capable of crossing the mitochondrial membrane and reaching cytosolic targets, resulting in several outcomes: activation of the nuclear factor-kappa B (NF-κB) pathway (which subsequently enhances the transcription of genes implicated in inflammation), synthesis of pro-inflammatory cytokines and activation of the inflammasomes, particularly the NLRP3 inflammasome (57, 58). ROS can also promote the release of calcium from the endoplasmic reticulum, which subsequently amplifies ROS production (56). PTH may contribute to alterations in the inflammatory profile of patients with PHPT as well. Immune cells (e.g. T cells), osteoblasts and osteoclasts express PTH receptors (59), their stimulation leading to activation of signaling pathways that can promote the release of growth factors and pro-inflammatory cytokines such as IL-1, IL-6, or TNF-α (60). Additionally, *in vitro* studies suggest that PTH can activate the NADPH oxidase (61) contributing to ROS generation, particularly superoxide, in the cytoplasm. This process exacerbates oxidative stress and its impact on the immune function. **Figure 2** illustrates the complex interplay between oxidative stress and chronic inflammation in PHPT.

IL-6 is a cytokine produced by immune cells (lymphocytes, monocytes), as well as other cell types, including hepatocytes, endothelial cells, osteoblasts or parathyroid cells (62, 63). It plays a key role in the acute phase response but is involved in regulating bone homeostasis as well and can act systemically to stimulate the hypothalamic-pituitary-adrenocortical axis (64). Circulating levels were found to be increased in patients with PHPT in the majority of studies, albeit not all. Correlations between IL-6 and PTH have also been identified in two of the studies (27, 28), although IL-6 was not different in the overall PTHP group compared with controls (27). These observations are supported by experimental data which showed that PTH can induce IL-6 production in osteoblasts (65), hepatocytes, Kupffer cells and endothelial cells (66) and that the parathyroid glands could be an important source of IL-6 as well, especially in conditions associated with parathyroid hyperplasia (63). The link between IL-6 and bone resorption parameters identified in two of the studies reinforces the role of this cytokine in the regulation of bone metabolism (28, 29). Furthermore, Nakchbandi et al. were able to establish cut-off values for IL-6 and its soluble receptor that might identify subjects with significantly greater rates of bone loss at the total femur (29). Assessment of IL-6 levels after parathyroidectomy yielded conflicting results. The different intervals between surgery and evaluation, ranging from one hour to 22 months could explain these discrepancies.

IL-11, a member of the IL-6 family of cytokines, participates in various physiological processes, including bone remodeling. It regulates osteogenesis, supporting proper bone formation and facilitating fracture healing, but also contributes to bone resorption by promoting the differentiation of osteoclast precursors into mature osteoclasts and increasing their activity (67). Nakchbandi et al. found a strong negative correlation between circulating levels of PTH and serum IL-11 and showed that parathyroidectomy led to an increase in IL-11. These observations suggest that PTH may play a role in modulating IL-11, potentially through an indirect mechanism. This is supported by the inverse relationship between IL-6 and IL-11 in response to PTH identified by the authors, leading to the conclusion that IL-6 inhibits IL-11 production (36).

TNF-α is one of the earliest and most potent pro-inflammatory cytokines released during an immune response to infection or injury. It is primarily produced by immune cells (macrophages, T-cells), but also by other cell types, including fibroblasts, endothelial cells or adipocytes (68). TNF-α levels were shown to be elevated in PHPT patients and either remained unchanged or decreased following parathyroidectomy. These findings are consistent with experimental data showing that T cells produce TNF-α in response to PTH stimulation (69).

Hs-CRP is a more precise test than CRP, elevated levels being linked to atherosclerosis and CV disease (70), type 2 diabetes, metabolic syndrome (71), autoimmune disorders (72) and cancer (73). As systematically analyzed here, Hs-CRP levels were found to be elevated in PHPT patients compared with controls. Additionally, correlations with PTH (25) and calcium (27) were observed. However, most studies that evaluated CRP found similar levels between PHPT patients and controls. These discrepancies might be explained by the fact that CRP tests assess relatively higher levels of inflammation (typical for conditions like infections, autoimmune diseases, or trauma), while Hs-CRP is much more sensitive and can detect very low levels of inflammation. While most of the studies did not reveal any significant changes in CRP levels after parathyroidectomy, Almqvist et al. (38) showed an increase in Hs-CRP one year following surgery and Christensen et al. (46) reported a rise in CRP levels at 6 months postoperatively. These changes could be attributed to tissue injury resulting from the surgical procedure.

Additionally, an increased level of interleukin-2 receptor (IL-2R) was observed in patients with PHPT (31). This receptor plays a key role in the regulation of immune responses, by influencing the proliferation and activity of immune cells, particularly T cells. IL-2 binds to the high-affinity IL-2R on activated T cells, stimulating their proliferation and promotes the differentiation of CD4+ helper T cells and CD8+ cytotoxic T cells (74).

IL-17A, a cytokine produced by T helper 17 (Th17) cells, is believed to be a key mediator of chronic inflammation (75, 76). It exerts its effects through binding to its specific receptor (IL-17R) on various cells including osteoblasts, osteocytes and osteoclast precursors, enhancing RANKL production and upregulating receptor activator of NF-κB (RANK), thereby facilitating osteoclast differentiation and bone resorption (77–80). PTH was found to be positively correlated with IL-17 in a study that included female students with vitamin D deficiency (81). Yankova et al. (47) showed that IL-17A levels were increased in patients with PHPT, an observation which is in accordance with data provided by Li et al. (82) showing that PHPT upregulates IL-17A mRNA expression in peripheral blood cells, an effect that is reversed following parathyroidectomy. On the other hand, Danzio et al. (48) did not observe any differences in IL-17A levels between PHPT patients and controls. IL-17A might promote the effects of abnormally high PTH on osteoclasts considering that IL-17R gene silencing or neutralization of IL-17A with monoclonal antibodies blunted the ability of PTH to induce bone resorption, while bone formation was not influenced (82, 83). However, in the two studies evaluating IL-17A that were included in the present review, RANKL and OPG values were similar in the PHPT and control groups (47, 48). Yankova et al. suggested that this finding might indicate that the RANK/RANKL/OPG system is not the sole regulator of bone metabolism in PHPT, while also highlighting that circulating concentrations of these molecules may not accurately represent the dynamics occurring within the bone microenvironment (47).

IL-10 is a cytokine with potent anti-inflammatory effects that plays a crucial role in regulating immune responses and maintaining tissue and cellular homeostasis (84, 85). Data regarding the interplay between PTH and IL-10 are conflicting. Experimental evidence indicated that intermittent administration of PTH resulted in increased IL-10 production in old rats. In a study that included patients with rheumatoid arthritis, a significant positive correlation between PTH and IL-10 was identified (86), while Ori et al. showed that in patients undergoing chronic dialysis, higher IL-10 levels were associated with lower PTH values (87). In PHPT patients, Meng et al. (50) identified similar levels of IL-10 in patients and controls, and no significant changes following parathyroidectomy.

The WBC was similar in PHPT patients and controls, while significant differences between the two groups were found for other markers derived from the FBC (NLR, PLR or RDW). A possible explanation could be that while the WBC measures the total number of white blood cells in the blood, it does not provide detailed information about the proportions of different types of immune cells (e.g., neutrophils, lymphocytes, monocytes). A chronic inflammatory state may contribute to ineffective erythropoiesis causing immature erythrocytes to enter the circulation, resulting in elevated RDW (88). Thus, NLR, PLR, RDW represent markers of inflammation that might reflect more subtle or chronic aspects of inflammation and immune dysregulation in diseases where low grade inflammation might not induce changes in the WBC but could shift the balance of immune cells or alter the erythropoiesis. Both NLR and PLR were shown to be correlated with IL-6, TNF-α, and CRP levels in studies performed on dialysis patients (89, 90). Also, NLR, PLR and RDW were found to be correlated with PTH (25, 30, 43). Regarding the changes in levels of helper T cells, suppressor T cells and the H/S ratio (helper T cells count divided by suppressor T cells count) conflicting results were found in the two studies that evaluated these markers (32, 34) and the different sample sizes for the study groups might account partially for these discrepancies. On the other hand, both studies revealed an altered response of T cells to lectins (PHA, concanavalin A), which was restored to normal following parathyroidectomy.

MHR is an emerging composite biomarker that reflects the interplay between inflammation and oxidative stress, with higher values being associated with increased cardiovascular risk, particularly in individuals with chronic kidney disease (91, 92). The divergent findings observed in the two studies assessing this marker may be attributed to differences in the duration of post-parathyroidectomy follow-up, with one study evaluating outcomes at 1 month (52), and the other over a longer period, ranging from 7 months to 2 years (53).

Fibrinogen, a key protein involved in blood clotting, wound healing, and immune responses, was also found to be elevated in PHPT patients and was correlated with the volume of parathyroid adenomas. Christensen et al. analyzed mRNAs extracted from samples of subcutaneous fat tissue from the cervical region and observed significant changes in the expression of genes associated with inflammation and metabolism in PHPT patients compared with controls (93). Based on these findings, the authors selected several serum markers associated with inflammatory processes (MMP9, RAGE, sCD-14, S100A4, and S100A8/A9) (94–98) and revealed that MMP9, sCD-14 and S100A4 were significantly elevated in PHPT patients. They also showed that both sCD-14 and S100A4 decreased following surgery, concluding that PHPT was characterized by an increased inflammatory state and parathyroidectomy provided a partial reversal of the systemic inflammation (42). In a different study, Christensen et al. examined systemic markers of IFNγ-mediated immune activation, as IFNγ pathways are known to be implicated in the development of several disorders (99–101). The authors observed that the K/T ratio was elevated in PHPT patients, suggesting that IFNγ-mediated cellular immune activation could be a characteristic feature of PHPT as well (46).

MCP-1 is a chemokine with a pivotal role in orchestrating immune responses by promoting the recruitment and infiltration of monocytes and macrophages, as well as facilitating the migration, activation, and differentiation of lymphocytes and natural killer cells (102, 103). It is highly expressed in the adipose tissue (104) and levels were found to be elevated in patients with complications associated with atherosclerosis (myocardial infarction or ischemic stroke) (105) and in individuals with obesity (106). Patients with PHPT exhibited higher levels of MCP-1 compared with control subjects (50). Furthermore, both studies that evaluated this parameter (49, 50) revealed a decrease after parathyroidectomy and positive correlations between PTH and MCP-1. These observations are in line with data from the literature. For instance, Sukumar et al. demonstrated that female individuals with high PTH have elevated levels of MCP-1, irrespective of adiposity (107). Moreover, experimental data from a rat model revealed that PTH administration led to a significant increase in serum MCP-1 concentrations, while also promoting osteoblastic expression of this chemokine (108). In human osteoblasts, parathyroid hormone-related protein (PTHrP) increased MCP-1 production as well (109). The effects of PTH on bone remodeling are dependent on the pattern of administration, with intermittent exposure eliciting anabolic effects and continuous exposure resulting in catabolic outcomes. This variability may be partially attributable to differential regulation of MCP-1 production by PTH, characterized by a moderate yet sustained increase under continuous administration, in contrast to a transient but pronounced upregulation following intermittent administration (110).

Evaluating chronic inflammatory markers in patients with PHPT could have significant clinical implications by providing valuable insights into the underlying pathophysiology of the disease and its classical and non-classical manifestations and complications. For instance, the prevalence of CV disease and hypertension is higher in PHPT patients compared to the general population, with several studies reporting improvements in cardiovascular outcomes following parathyroidectomy (111, 112). Chronic inflammation has been closely linked to key pathological processes underlying CV disease, including endothelial dysfunction, arterial stiffness, and atherosclerosis (113–116). Pro-inflammatory stimuli are thought to contribute to the initial stages of atherosclerosis by altering the expression of adhesion molecules which mediate the attachment of circulating lymphocytes and monocytes to the endothelial cells (113). Therefore, nowadays, atherosclerosis is no longer regarded solely as a disorder of lipid storage, but rather a diffuse disease, with inflammation playing a central role in its development. Several markers known to be associated with CV disease, including IL-6, TNF-α, Hs-CRP, fibrinogen and MCP-1 were found to be increased in PHPT patients in this review. This suggests that the chronic inflammation in PHPT could play a significant role in the development of CV complications in these individuals. Consequently, evaluating inflammatory status may offer valuable insights for improving cardiovascular risk stratification in PHPT patients.

**Figure 3:**
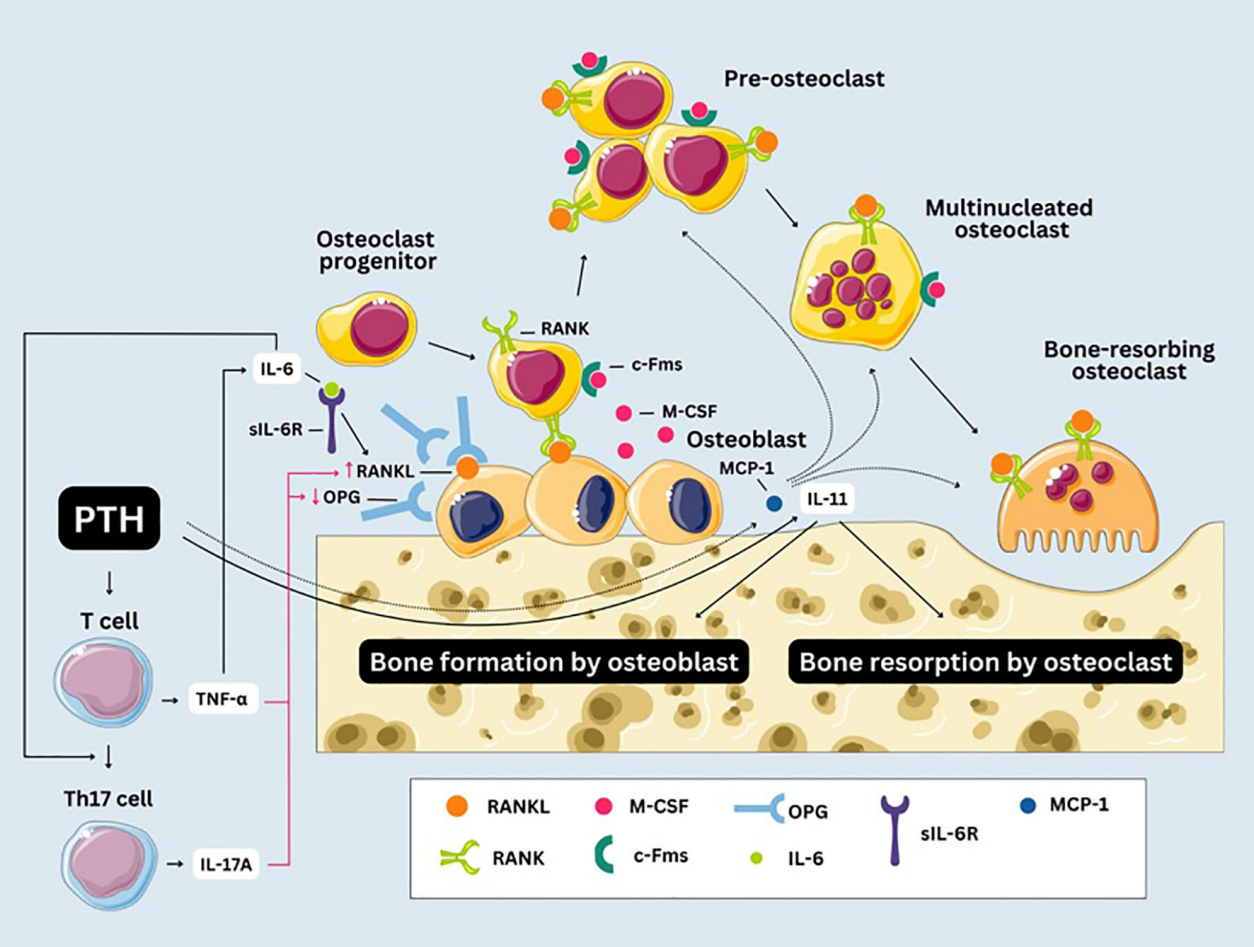
Chronic inflammation and bone resorption in primary hyperparathyroidism. Macrophage Colony-Stimulating Factor (M-CSF) interacts with the Colony-Stimulating Factor-1 Receptor (c-Fms) on progenitor cells ensuring their commitment to the osteoclast lineage. Mononuclear precursors undergo fusion to generate pre-osteoclasts, which subsequently fuse with one another to form multinucleated syncytia. After fusion, multinucleated osteoclasts undergo further differentiation into bone-resorbing osteoclasts. RANK, receptor activator of NF-κB; RANKL, receptor activator of NF-κB ligand; OPG, osteoprotegerin; sIL-6R, IL-6 soluble receptor; MCP-1, monocyte chemoattractant protein-1.

Furthermore, chronic inflammation might also be involved in the PTH-induced bone remodeling, considering that levels of factors derived from mononuclear and bone cells that promote bone resorption, including IL-6, sIL-6R, IL-11, TNF-α or MCP-1, were shown to be altered in PHPT patients. There are several mechanisms through which chronic inflammation might induce bone resorption in patients with PHPT (**Figure 3**). PTH stimulates osteoblasts to produce IL-6, which acts synergistically with its soluble receptor to stimulate osteoclastic bone resorption by promoting the expression of RANKL on osteoblast surfaces, which then binds to RANK on osteoclast progenitors, triggering their differentiation into osteoclasts (65, 117, 118). The role of IL-6 and its receptor in bone resorption is supported by data showing that their serum levels correlate with rates of bone turnover and predict rates of bone loss in patients with PHPT (28, 29). T cells might also significantly contribute to PTH-induced bone resorption by secreting TNF-α in response to PTH stimulation (69, 119). TNF-α stimulates osteoblasts to produce IL-6 (120), and both TNF-α and IL-6 are involved in promoting the differentiation of T cells into Th17 helper cells, which release IL-17A (69). Li et al. (82) revealed that in murine models, continuous PTH (cPTH) administration promotes the differentiation of Th17 cells, while also increasing the responsiveness of naive CD4^+^ T cells to TNF. TNF-α and IL-17A enhance RANKL production while TNF-α also suppresses the secretion of OPG (a protein that functions as a soluble decoy receptor for RANKL, binding to it and preventing the formation of the RANKL-RANK complex), thus creating a favorable environment for osteoclastic bone resorption (69). The finding that in mice lacking TNF-α production from T cells, PTH fails to induce bone resorption underlies the importance of TNF-α in this process (121). Furthermore, the osteoclastic expansion induced by PTH can be blocked by silencing the PTH receptor 1 (PR) in T cells, indicating that PR signaling in T cells plays an important role in PTH induced bone loss (121). MCP-1, secreted by osteoblasts in response to PTH, might also play a key role in bone resorption. cPTH in a rodent model led to an increase in both MCP-1 and RANKL and the authors hypothesized that MCP-1 may enhance RANKL-driven bone resorption by attracting preosteoclasts and osteoclasts to sites of bone remodeling and supporting their differentiation (122). Moreover, it was shown that deletion of the MCP-1 gene could potentially mitigate the catabolic impact of cPTH on the bone, by impairing the recruitment of monocytes, macrophages, and osteoclasts (123). Modulation of IL-11 by PTH might be another mechanism involved in the alterations in bone homeostasis seen in PHPT patients (67). Evaluating inflammatory markers might therefore be useful in identifying patients at heightened risk for osteoporosis and fragility fractures, beyond what can be predicted by PTH and calcium levels alone.

A significant number of patients with PHPT experience vague symptoms, including fatigue, weakness, mood disturbances like depression and anxiety, cognitive difficulties, or sleep disturbances (124–126). Growing evidence indicates that inflammation plays a role in various neurological and psychiatric disorders (127–129). Hence, chronic inflammation could partially explain these nonspecific symptoms in patients with PHPT and inflammatory biomarkers could help predict which patients are more susceptible to these symptoms, allowing for early mental health support and management.

Ultimately, by assessing inflammatory markers in PHPT patients, clinicians can potentially identify individuals at higher risk for severe disease progression and comorbidities, allowing for more tailored therapeutic interventions. Moreover, if chronic inflammation is proven to play a key pathogenic role in PHPT, it could substantiate the potential for incorporating anti-inflammatory or immunomodulatory therapies as adjuncts to the surgical or medical management of the disease.

## Limitations

The most important limitations of this review include the high heterogeneity in markers that were evaluated, the variety of biological samples used and the small sample sizes of many of the reviewed studies. Another limitation involves the control groups that were used. Firstly, not all of them included healthy individuals, thus the associated diseases might account at least partially for the changes observed in the biomarkers. The lack of matching for BMI in some studies could also yield conflicting results. This is due to the fact obesity is a disease with an important inflammatory component, as adipose tissue, especially visceral fat, is metabolically active and plays a key role in the body’s inflammatory response. At the same time, the timespan between parathyroidectomy and biomarker assessment was highly variable, possibly leading to interferences, as surgery itself is known to be involved in increasing inflammation. We opted not to conduct a meta-analysis due to the considerable clinical, biochemical and methodological heterogeneity among the studies, which made a reliable quantitative analysis unfeasible. Nevertheless, we believe that the systematic review offers substantial value to the field by providing a comprehensive synthesis of findings on chronic inflammation markers in PHPT across diverse study contexts, underscoring key methodological challenges, while also highlighting the clinical relevance of inflammatory markers assessment, and offering insights into potential pathophysiological mechanisms underlying PHPT and its associated complications.

## Future perspectives

Understanding the role of inflammation in PHPT opens up numerous avenues for future research. Exploring how inflammation contributes to the various symptoms and complications of PHPT could provide insights into the disease’s mechanisms and progression. A second research area could include exploring the potential benefits of anti-inflammatory agents as therapeutic instruments, especially in patients who are not candidates for surgery. Another potential future direction involves the identification of markers with prognostic significance for treatment response or those that could contribute to the distinction between parathyroid adenoma, hyperplasia and carcinoma preoperatively, thus facilitating the decisions regarding the surgical approach.

## Conclusion

To conclude, patients with PHPT exhibit several alterations in the pathways associated with chronic inflammation. These changes could be involved in the pathogenesis of the disease and might also account for some of the complications associated with PHPT including bone resorption, CV disease or neuropsychiatric disorders. By elucidating these pathways, researchers and clinicians can develop targeted interventions to improve patient outcomes, reduce complications, and ultimately enhance the quality of life for individuals affected by this condition.

## Data Availability

The original contributions presented in the study are included in the article/Supplementary Material. Further inquiries can be directed to the corresponding author.
